# A feedback loop between DNA damage, genomic instability, and cytoplasmic DNA sensing contributes to cytokine production in COVID-19

**DOI:** 10.1007/s00705-025-06383-6

**Published:** 2025-08-11

**Authors:** Miguel A. Fernández-Rojas, Ana María Salazar, Patricia Ostrosky-Wegman, Ana Flisser, Fela Mendlovic

**Affiliations:** 1 Departamento de Microbiología y Parasitología, Facultad de Medicina, Universidad Nacional Autónoma de México (UNAM), Mexico City, Mexico; 2 Departamento de Medicina Genómica y Toxicología Ambiental, Instituto de Investigaciones Biomédicas, Universidad Nacional Autónoma de México (UNAM), Ciudad Universitaria, Apartado Postal 70228, Mexico City, Mexico; 3 Facultad de Ciencias de la Salud, Universidad Anáhuac México Norte, Huixquilucan, Estado de Mexico Mexico

**Keywords:** Micronucleus, cGAS-STING, AIM2, NLRP3, Cytokine storm, COVID-19

## Abstract

Since the onset of the COVID-19 pandemic, several studies have investigated the inflammatory responses triggered by SARS-CoV-2 infection. In 2021, it was proposed that the cytokine storm observed in patients with severe COVID-19 may be initiated by sensing of cytoplasmic DNA released by micronuclei, which arises as a consequence of virus-induced genomic instability. Subsequent studies have described the presence of micronuclei and other genotoxic and cytotoxic markers in COVID-19 patients. However, the association between the development of a cytokine storm and cytoplasmic DNA sensing remains to be fully elucidated. In this review, we summarize current evidence on the dysregulated cytokine production in response to the detection of genetic material during SARS-CoV-2 infection. We focused mainly on the dysregulated production of cytokines induced by the activation of cytosolic DNA sensing pathways that promote inflammation. We emphasize the need to analyze the contribution of these signaling complexes to COVID-19 pathophysiology. DNA sensing amplifies the inflammatory response and plays a crucial role in the pathogenesis of severe disease manifestations observed in infected patients. Understanding this complex interplay can provide insights into potential therapeutic targets aimed at mitigating the hyper-inflammatory responses seen in severe COVID-19 cases.

## Introduction

As COVID-19-related deaths, hospitalizations, and severe cases declined, the World Health Organization (WHO) declared the end of COVID-19 as a Public Health Emergency of International Concern in 2023. This measure prompted a shift toward long-term management of the virus. Nevertheless, the evolution of SARS-CoV-2, its interaction with the immune system, and its immune evasion potential are an ongoing concern [1, 2]. A subset of patients develop symptomatology and severe disease linked to the occurrence of a cytokine storm. Annual confirmed cases and deaths highlight the need to understand the mechanisms involved in the induction of this dysregulated immune response.

SARS-CoV-2 is a single-stranded, positive-sense RNA virus that can initiate immune recognition by pattern recognition receptors (PRRs) through RNA and protein recognition. In addition, SARS-CoV-2 infection can cause DNA damage and genomic instability. Cytoplasmic DNA is a well-known pathogen-associated molecular pattern (PAMP) and damage-associated molecular pattern (DAMP). It can originate from various sources, including viral infections, mitochondrial DNA rupture, release from micronuclei (MN), or phagocytosis of cell-free DNA (cfDNA) and nucleosomes or cell free chromatin particles (cfChPs) [3–7]. DNA fragments in the cytoplasm are detected by PRRs, which trigger immune responses to clear infections or damaged cells. This recognition is crucial for maintaining cellular integrity and initiating appropriate immune responses against pathogens or cellular damage. However, when dysregulated, it plays a crucial role in the pathogenesis of severe disease manifestations. Here, we review existing evidence that recognition of DNA during SARS-CoV-2 infection contributes to the generation of a cytokine storm and the immunopathology developed during COVID-19.

## DNA damage induced by SARS-CoV-2 infection

MN are extranuclear structures containing lagging chromosomes or chromosome fragments that are released into the cytoplasm because of DNA damage or mitotic errors. Their formation is linked to an impaired or incomplete DNA damage response (DDR), where unrepaired or missegregated chromosomal material becomes encapsulated in MN during anaphase. MN are frequently observed in pathologies and processes such as inflammation, senescence, and genotoxic stress [8, 9]. MN have a fragile nuclear envelope, predisposing them to rupture and exposure of their DNA content to the cytoplasm, activating downstream cytoplasmic DNA sensing and signaling pathways, cell cycle arrest, and cytokine production [9]. Additionally, cfDNA and cfChPs released by dying neighboring cells can enter the cell by endocytosis, triggering oxidative stress by mitochondrial damage and reactive oxygen species (ROS) production, and act as DAMPs that are identified by DNA sensing pathways [3, 10, 11].

The DDR is a complex signaling network activated by DNA damage, which leads to DNA repair to maintain genome stability. Upon DNA damage, several pathways are activated. Ataxia-telangiectasia mutated (ATM), ataxia-telangiectasia RAD3-related (ATR), and DNA-dependent protein kinase (DNA-PK) are key regulators of the DDR, repair signaling, and cell cycle arrest [12, 13].

DNA damage and genomic instability are well-documented consequences of viral infections, including SARS-CoV-2 infections, as evidenced by the formation of MN and the presence of cfDNA in infected cells and in circulation [14, 15]. A recent study showed that SARS-CoV-2 causes DNA damage and alters the DDR response by at least two mechanisms. First, two viral proteins, ORF6 and NSP13, induce the degradation of checkpoint kinase 1 (CHK1), an ATR-linked protein, through deoxynucleoside triphosphate shortage, leading to impaired DNA replication. Second, the virus interferes with the activation of damage-induced long non-coding RNAs, which compromises DNA repair and further exacerbates DNA damage. Loss of CHK1 in infected cells leads to the formation of γH2AX foci and MN that are positive for cyclic GMP-AMP synthase (cGAS) staining, as well as increased pro-inflammatory cytokine production and cellular senescence through the activation of pro-inflammatory pathways, cGAS/stimulator of interferon genes (STING), signal transducer and activator of transcription (STAT)1, and p38/mitogen-activated protein kinase (MAPK) [16]. Therefore, enhancing DDR activation may represent a potential strategy to mitigate the excessive inflammation triggered by SARS-CoV-2 infection, as inhibition of ATR has been shown to reduce levels of the pro-inflammatory cytokine IL-6 [17].

cfDNA originating in multiple organs, including the kidney, heart, and lungs, has been detected in serum samples from COVID-19 patients [18], and the authors reported a correlation between cfDNA and inflammatory biomarkers, such as C-reactive protein (CRP) or D-dimer, as well as the overproduction of ROS by the self-sensing of cfDNA, suggesting an inflammatory feedback loop. Moreover, patients who develop severe disease show higher levels of cfDNA in their plasma than those with milder disease [19]. In addition, cfChPs have been associated with COVID-19 severity [20]. Thus, emerging evidence suggests that COVID-19 is associated with the induction of DNA damage, which in turn activates cytoplasmic DNA sensing pathways and promotes inflammatory signaling.

## Cytoplasmatic DNA sensing as a pro-inflammatory stimulus in SARS-CoV-2 infection

Cytoplasmic DNA that accumulates due to a failed DDR or endocytosed cfDNA is sensed by innate immune receptors– primarily Toll-like receptor (TLR)−9, cGAS, and absent in melanoma 2 (AIM2) [21–25]. Upon sensing of DNA, TLR-9 in endosomes recruits MyD88 to induce expression of IRF7, IRF1, and NF-κB, leading to the expression of type I interferon (IFN), interferon-stimulated genes (ISGs), and pro-inflammatory cytokines, respectively. In addition, cytoplasmic DNA sensing can be initiated by cGAS, which catalyzes the synthesis of 2′3′-cyclic GMP-AMP (cGAMP). cGAMP subsequently binds to STING, triggering downstream signaling [26–28]. The cGAS-cGAMP-STING pathway activates IRF3/7, type I IFN, and NF-κB signaling, resulting in the production of pro-inflammatory cytokines [29, 30]. The binding of cGAS to the DNA backbone is nonspecific. Thus, endogenous DNA that leaks from MN or exogenously endocytosed cfDNA can activate the cGAS-STING axis. Furthermore, type I IFN production can be stimulated by the recognition of mitochondrial DNA (mtDNA) released from endothelial cells and engulfed by macrophages, triggering the production of pro-inflammatory cytokines through the cGAS-STING pathway [31]. This activation can initiate immune responses against pathogens but may also contribute to inflammation-driven pathologies [32–34].

AIM2 is a double-stranded (dsDNA) sensor that, upon binding, drives inflammasome assembly and subsequently activates caspase 1, which cleaves pro-IL-1β, pro-IL-18, and gasdermin D (GSDMD) [35]. GSDMD forms pores in the plasma membrane and triggers pyroptosis, an inflammatory form of cell death. Activated IL-1β and IL-18 are released through the GSDMD pores [36]. AIM2 expression has been reported to be elevated in severe COVID-19 patients [37]. Specifically, AIM2 has been found to be overexpressed in nasopharyngeal epithelial cells and peripheral blood mononuclear cells (PMBCs) from COVID-19 patients and to correlate with disease severity. The presence of AIM2 in circulating PMBCs suggests systemic immune cell activation [38]. Indeed, infected SARS-CoV-2 monocytes have been shown to undergo AIM2 and NLPR3-inflammasome-mediated pyroptosis and cytokine release, contributing to COVID-19 immunopathology [39].

The NOD-, LRR-, and pyrin-domain-containing protein 3 (NLRP3) inflammasome is activated by multiple upstream signals, including mitochondrial dysfunction, ROS generation, pore formation, K^+^ efflux, and activation of P2X7 receptors by extracellular ATP released after pyroptosis. While NLRP3 does not bind DNA directly, its activation can be promoted indirectly by mtDNA release, often in the context of STING-mediated stress responses, ultimately leading to production of pro-inflammatory cytokines [40]. NLRP3 inflammasome activation promotes the maturation and secretion of IL-1β and IL-18 and induces pyroptosis through GSDMD pore formation [24, 41–44]. In the context of COVID-19, NLRP3 inflammasome activation has been observed in patients and associated with pyroptosis of infected monocytes [45, 46]. Moreover, production of cleaved GSDMD has been linked to an increased risk of severe COVID-19, and its presence has been confirmed in lung tissue and bronchoalveolar lavage fluid of affected patients [47, 48]. NLRP3 can be activated by SARS-CoV-2 viroporin 3a through two main mechanisms: mitochondrial damage leading to DNA damage by ROS production and disruption of the intracellular concentration of K^+^ ions. This cascade of events results in the production of pro-inflammatory cytokines IL-1β and IL-18 [49]. COVID-19 patients show activation of NLPR3, maturation and release of pro-inflammatory cytokines, aberrant production of mitochondrial superoxide (a source of ROS), and lipid peroxidation. The persistent aberrant inflammatory response found in some patients reinforces the idea that pathological inflammation is partly responsible for long-term COVID-19 [50].

Recent studies have suggested that the detection of endogenous DNA resulting from cellular damage induced by SARS-CoV-2, endocytosis of free dsDNA, cell-free chromatin (cfCh), and cfChPs released by neighboring apoptotic cells can contribute to the dysregulated cytokine production observed in severe COVID-19 patients [20, 51–53]. Additionally, *in vitro* studies using HeLa cells have demonstrated that syncytium formation via angiotensin-converting enzyme 2 (ACE2)/spike (S) protein interaction induces MN production, DNA damage induction, and cGAS-STING pathway activation [54]. This pathway triggers type I IFN production, which has been linked to inflammation and poor outcomes in severe COVID-19 cases [55–57].

**Fig. 1:**
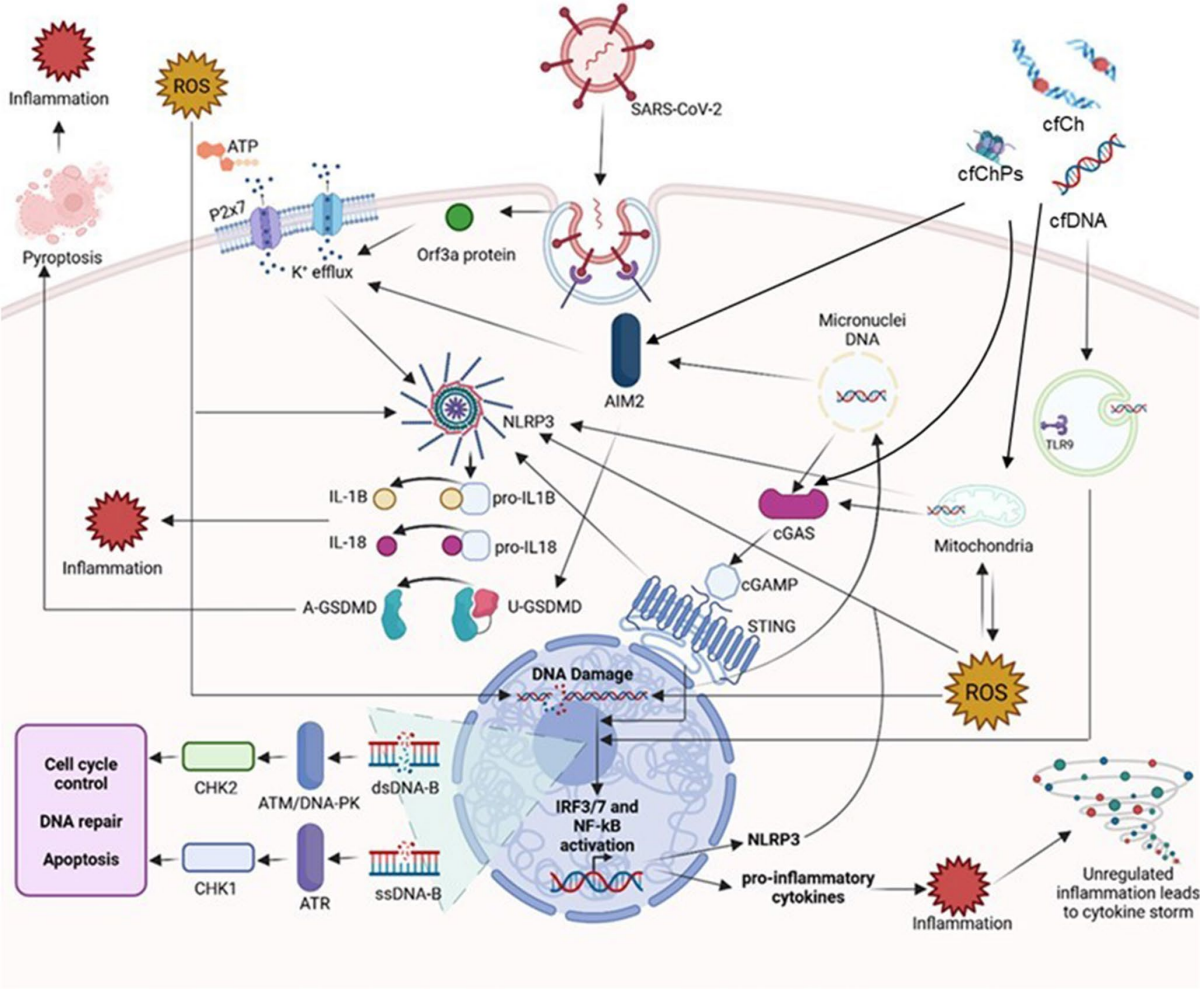
Potential pro-inflammatory response triggered by the persistent detection of cytoplasmic DNA in severe COVID-19. After internalization of viral material, immune cells can produce reactive oxygen species (ROS), potentially damaging host DNA and activating the ATR/ATM pathways. This leads to genomic instability, cell cycle dysregulation, DNA repair, and/or apoptosis. cfChPs can produce ROS by mtDNA damage. A feedback loop between mtDNA damage induced by ROS and ROS overproduction by mitochondrial dysfunction activates the NLRP3 and AIM2 inflammasomes, promoting IL-1β and IL-18 maturation and cell death with pro-inflammatory effects (pyroptosis) by activation of gasdermin D (GSDMD). NLRP3 activation can also be triggered by SARS-CoV-2 viroporins, P2X7/ATP, mtDNA, or self-DNA signaling through AIM2. Additionally, cGAS-STING activity can be induced by cfCh, mtDNA and nuclear DNA from MN released to the cytoplasm. The endosomal receptor TLR-9 can detect self-mtDNA and cfDNA from neighboring apoptotic cells. All of these signaling events lead to IRF and NF-κB pathway activation and overproduction of several cytokines and chemokines, promoting overwhelming inflammation

Therefore, we propose an inflammatory feedback loop in which SARS-CoV-2, through the action of various accessory proteins and the induction of cytotoxic processes such as karyorrhexis and karyolysis, promotes the release of cfDNA, mtDNA, cfCh, and cfChPs into the cytoplasm. The accumulation of cytoplasmic DNA stimulates the activation of innate immune sensors, including TLR-9, the cGAS-STING pathway, and AIM2, that lead to the production of ROS, further DNA damage, and the potential formation of MN. Recognition of DNA by these pathways triggers the production of pro-inflammatory cytokines via the IRF3 and NF-κB signaling cascades and promotes the synthesis of NLRP3 components. Activation of the NLRP3 and AIM2 inflammasomes leads to the maturation and secretion of IL-1β and IL-18, as well as the induction of pyroptosis. Furthermore, K^+^ efflux mediated by SARS-CoV-2 viroporin 3a activity, ATP/P2x7 receptor interaction, and AIM2 activation, provides additional signaling that promotes NLRP3 inflammasome assembly. Collectively, these events establish an inflammatory feedback loop during SARS-CoV-2 infection, linking DNA damage, cytoplasmic DNA release, and the induction of a proinflammatory state derived from DNA detection by innate immune recognition. This loop functions as an amplifying circuit, causing excessive immune activation and the immunopathology associated with SARS-CoV-2 infection (Fig. 1).

The critical role of cytosolic nucleic acid sensing in driving excessive inflammation and immunopathology in critical illnesses has been extensively reviewed by Chen et al. [58] and provides conceptual support for our proposed model. Our proposal specifically highlights the SARS-CoV-2-mediated self-amplifying inflammatory feedback loop initiated by viral-induced DNA damage, cytoplasmic DNA release, and innate immune activation [58]. Moreover, COVID-19 severity has been associated with several comorbidities, including metabolic disorders (obesity and diabetes) [59]. Current evidence describes an important role of cGAS-STING and NLRP3 as critical players in the development of these comorbidities [60–62]. Patients with metabolic disorders develop severe COVID-19 with exacerbated metabolic inflammation partially regulated by NLRP3 [63]. These findings suggest that infection by SARS-CoV-2 may amplify existing systemic inflammation in obese and diabetic patients by further activating NLRP3 [64, 65]. This inflammatory state could be activated by the detection of genetic material from SARS-CoV-2 replication or the DNA released into the cytoplasm due to DNA damage, genomic instability, and MN production induced by the infection [66, 67].

The association between higher serum levels of MN, cfDNA, cfCh, and cfChPs in severely ill COVID-19 patients suggests the potential use of genetic material as a predictor of critical illness in cases of SARS-CoV-2 infection [20–22, 68–71], similar to its proposed use as a biomarker for metabolic diseases and cancer [72, 73]. Some studies have evaluated the utility of cfDNA and cfChPs in plasma samples from COVID-19 patients as a marker for disease detection and outcome prediction [19, 20, 69]. The identification of a positive correlation between cfDNA or cfChPs and disease severity highlights the need for further research.

## Induction of cytokine storm by cytoplasmic DNA sensing in SARS-CoV-2 infections

Cytokine storm, also known as cytokine release syndrome (CRS), is characterized by excessive production of pro-inflammatory cytokines, leading to systemic inflammation and organ damage [74, 75]. Usually, a cytokine storm resolves once the infection ends. However, in severe cases, cytokine overproduction persists, inducing systemic damage, sepsis, and possible death [76, 77]. For example, in severe and critical COVID-19 cases, hyperactivation of the innate immune response leads to CRS development. Specifically, higher levels of IL-1β, IL-6, IL-8, IL-10, IL-18, and TNFα have been found in patients with severe disease and complications, such as acute respiratory distress syndrome (ARDS), which are the main cause of mortality in COVID-19 [78, 79]. Other consequences of the overwhelming production of cytokines are tissue damage, multiple organ failure, T-cell exhaustion, and secondary bacterial infections [80, 81].

Several cytokines induced by pathways activated by DNA sensing have been associated with SARS-CoV-2 infection. A report in 2022 showed that activation of the NF-κB pathway by cGAS-STING signaling induces the overproduction of several proinflammatory cytokines, particularly IL-6 and IP-10. Moreover, it was shown that inhibition of STING leads to a decrease in mRNA and protein levels of IL-6, associated with a reduction in the nuclear accumulation of p65/RELA dimer and inhibition of the NF-κB pathway [82]. Early studies indicated an association between higher levels of IL-6 and increased severity and mortality in COVID-19 patients; however, subsequent evidence has been inconsistent [83, 84]. For instance, a meta-analysis by Leisman et al. showed lower IL-6 levels in patients with severe COVID-19 than in those with sepsis or ARDS [85]. Similarly, a 2021 review showed that while high IL-6 levels were commonly reported in H1N1 influenza A virus and SARS-CoV-1 infections, this was not consistent in SARS-CoV-2 infection cases [75]. Accordingly, neutralization of IL-6 by a specific antibody failed to achieve the desired effect during a phase 3 randomized clinical trial (NCT04320615) [86], despite promising results observed in earlier stages [87, 88]. These observations suggest that IL-6 levels alone are not the only factor associated with disease outcome in COVID-19.

IL-18 levels increase during SARS-CoV-2 infection, primarily due to inflammasome activation [45, 89]. A comprehensive literature review comparing cytokine responses to different respiratory viruses revealed that SARS-CoV-2 induces a distinct cytokine profile, characterized by elevated levels of IL-18, unlike other coronaviruses or influenza A virus, where such an increase is not observed [90]. Activation of the NLRP3 inflammasome in response to SARS-CoV-2 infection has been observed in postmortem samples and peripheral blood mononuclear cells from COVID-19 patients [45]. Macrophages from infected individuals often exhibit a hyperactivated pro-inflammatory state, contributing to CRS. This hyperinflammatory state and the elevated levels of IL-18 have been associated with disease severity and poor outcomes in severe COVID-19 cases [91–94].

In a single-cell transcriptomic assay, Zhang et al. identified monocyte clusters enriched in IL-18, amphiregulin, and epiregulin expression [95]. These monocyte subsets displayed a pro-inflammatory transcriptional signature and were predominantly found in severe and critical cases in which the patient developed pulmonary fibrosis. The frequency of these monocyte populations increased with disease severity, suggesting their potential use as biomarkers of clinical progression. Collectively, these findings support a central role for IL-18 in COVID-19 pathogenesis and underscore its association with disease severity and adverse outcomes [91, 96, 97].

Additionally, the SARS-CoV-2 S protein has been shown to inhibit mitophagy leading to mitochondrial ROS accumulation, enhancing NF-κB phosphorylation and NLRP3/IL-18-mediated cardiopulmonary inflammation [89]. Consistent with these findings, NLRP3 inflammasome activation has been detected in tissue samples of COVID-19 patients with ARDS, in contrast to non-COVID-19 non-ARDS controls [98]. Given its immunopathogenic role, IL-18 may be considered as a promising biomarker of COVID-19 severity, and therapeutic strategies targeting IL-18 inhibition, such as neutralization with IL-18 binding protein, may offer clinical benefits in severe cases [89].

Although previous studies have shown a positive correlation between cfDNA, cytokine production, and disease progression in cancer and sepsis [99–101], only limited evidence links the sensing of cfDNA, cfCh, or cfChPs with the dysregulation of cytokines during SARS-CoV-2 infection. A recent study in solid-organ transplant recipients (SOTRs) with COVID-19 found a strong correlation between monocyte-derived cfDNA and pro-inflammatory cytokines, including IL-6 and IL-18. Elevated levels of donor-derived cfDNA in these patients were also associated with CRS-related cytokines, such as IL-8, IL-10, and IL-18 [102].

IL-1β expression has been associated with hyperactivated neutrophils in severe COVID-19 pneumonia cases [93]. In addition, monocytes from severe COVID-19 patients spontaneously secrete IL-1β when incubated *in vitro*, a response that is reversed upon treatment with an IL-1β antagonist. While the exact mechanism of NLRP3 inflammasome activation was not demonstrated in these studies, it seems plausible to speculate that cytosolic DNA sensing, in addition to the recognition of viral proteins, contributes to inflammasome activation and cytokine production during SARS-CoV-2 infection [103].

The precise mechanisms responsible for the pro-inflammatory effects of cfDNA and its contribution to cytokine dysregulation in patients with severe and critical COVID-19 remain to be fully elucidated. Nevertheless, current evidence suggests that cytoplasmic DNA sensing activates inflammatory pathways that drive cytokine production. These pathways, therefore, represent potential therapeutic targets for the control of cytokine storms induced by viral infections. The elevated levels of IL-1β and IL-18 observed in COVID-19 patients with CRS support the involvement of the DDR and DNA sensing, via downstream inflammasome activation, amplifying inflammation and contributing to tissue injury.

In contrast to the commonly accepted models and evidence associating higher levels of pro-inflammatory cytokines, genotoxicity, and cytotoxicity with severe COVID-19 [78, 104, 105], recent studies show that mild cases may exhibit unexpectedly higher levels of IL-1β, IL-18, IL-6, MCP-1, and IL-4, as well as specific IgM and IgG anti-SARS-CoV-2 antibodies, compared to severe cases [106, 107]. Moreover, markers of cellular damage such as karyorrhexis and karyolysis have been reported to be higher in mild cases than in severe cases [106, 108]. These contrasting findings underscore the importance of investigating the possible association between the induction of genotoxic and cytotoxic damage and CRS or the modulation of individual cytokines. A better understanding of the complex interplay between DNA damage, cytokine responses, and disease severity is necessary for distinguishing mechanisms that lead to protective immunity from those that drive immunopathology.

## Conclusion

The critical role of endogenous and exogenous DNA detection is important in triggering immune and inflammatory responses during SARS-CoV-2 infection. The intrinsic production of DNA fragments (DNA leaked from MN, cfDNA, or cfChPs), DNA released by dying neighboring cells, and DNA damage and sensing, result in a feedback loop during viral infection, which triggers the production of predominantly pro-inflammatory cytokines. When properly controlled, the infection resolves. However, a lack of proper regulatory mechanisms can result in an undesired cytokine storm. It would be especially interesting to continue investigating the role of IL-18, given that elevated levels of this cytokine are associated with disease severity and that the pattern of cytokine expression in COVID-19 differs for those observed with other respiratory viruses, including other coronaviruses. The NLRP3 and AIM2 inflammasomes and the cGAS-STING pathway are critical links between DNA recognition and induction of the immune response against SARS-CoV-2. Inhibition of these cytosolic sensors might be an efficient way of reducing excessive inflammation and developing prevention strategies aimed at reducing the risk of severe or critical clinical outcomes. Nevertheless, the timing of interventions must be considered, as early activation of the immune response is necessary to limit infection, followed by dampening of the antiviral response after the infection is controlled in order to prevent immunopathology. Given the relevance of DNA sensing in disease progression and as an amplifier of the immune response, further studies are necessary to evaluate the potential for using DNA damage as a biomarker of disease outcome and a potential target for drug therapy. Understanding and controlling the signaling pathways activated by DNA sensing during SARS-CoV-2 infection can provide better management guidelines and benefits for patients.
